# Characterization of the Complete Chloroplast Genome of *Acer truncatum* Bunge (Sapindales: Aceraceae): A New Woody Oil Tree Species Producing Nervonic Acid

**DOI:** 10.1155/2019/7417239

**Published:** 2019-11-24

**Authors:** Qiuyue Ma, Yanan Wang, Lu Zhu, Changwei Bi, Shuxian Li, Shushun Li, Jing Wen, Kunyuan Yan, Qianzhong Li

**Affiliations:** ^1^Institute of Leisure Agriculture, Jiangsu Academy of Agricultural Sciences, Nanjing 210014, China; ^2^The Southern Modern Forestry Collaborative Innovation Center, Nanjing Forestry University, Nanjing, China; ^3^School of Biological Science and Medical Engineering, Southeast University, Nanjing 210037, China

## Abstract

*Acer truncatum*, which is a new woody oil tree species, is an important ornamental and medicinal plant in China. To assess the genetic diversity and relationships of *A. truncatum*, we analyzed its complete chloroplast (cp) genome sequence. The *A. truncatum* cp genome comprises 156,492 bp, with the large single-copy, small single-copy, and inverted repeat (IR) regions consisting of 86,010, 18,050, and 26,216 bp, respectively. The *A. truncatum* cp genome contains 112 unique functional genes (i.e., 4 rRNA, 30 tRNA, and 78 protein-coding genes) as well as 78 simple sequence repeats, 9 forward repeats, 1 reverse repeat, 5 palindromic repeats, and 7 tandem repeats. We analyzed the expansion/contraction of the IR regions in the cp genomes of six *Acer *species. A comparison of these cp genomes indicated the noncoding regions were more diverse than the coding regions. A phylogenetic analysis revealed that *A. truncatum *is closely related to *A. miaotaiense*. Moreover, a novel *ycf4-cemA* indel marker was developed for distinguishing several *Acer *species (i.e., *A. buergerianum*, *A. truncatum*, *A. henryi*, *A. negundo*, *A. ginnala*, and *A. tonkinense*). The results of the current study provide valuable information for future evolutionary studies and the molecular barcoding of *Acer* species.

## 1. Introduction

*Acer truncatum *Bunge, which is a member of the order Sapindales and the family *Aceraceae*, is a new versatile oil-producing woody tree that is widely distributed in northern China, Korea, and Japan, where it is a native species, but it has also been detected in Europe and North America [1]. This tree species represents a potential source of medicinal compounds. Many highly bioactive compounds have been extracted from *Acer *species, such as flavonoids, tannins, alkaloids, and terpenoids [2]. *Acer truncatum* seeds are processed to extract the seed oil, which was listed as a new food resource by the Ministry of Health of the People's Republic of China in 2011. Approximately 5–6% of the *A. truncatum *seed oil is nervonic acid (C24 : 1) [3]. Nervonic acid, which is a key component of brain nerve cells and tissues, promotes the repair and regeneration of nerve cells and damaged tissues, and has been detected in the seed oil of a number of plants. Thus, *A. truncatum *seed oil represents a novel plant resource with potential applications for treating human cerebral and neurological problems [4].

Chloroplasts (cps) have important functions related to some essential metabolic pathways, including photosynthesis and glycometabolism [5, 6]. In plants, the DNA-replication mechanism associated with the cp genome is independent of the nuclear DNA-replication mechanism. Moreover, the cp genome is more highly conserved than the nuclear genome. In 1986, the liverwort (*Marchantia polymorpha*) cp genome became the first such genome to be described [7]. The subsequent emergence of rapid and cost-effective genome-sequencing technologies has led to more cp genomes being sequenced, with the resulting data deposited in the GenBank database. These sequences indicate that the angiosperm cp genomes typically form a circular DNA molecule comprising 120–170 kb that encode 120–130 genes [8]. The circular cp genome structure consists of the following four segments: two inverted repeat (IR) regions separated by large single-copy (LSC) and small single-copy (SSC) regions [9, 10]. However, genome size variations [11], rearrangement events [12–14], and gene losses [15] have been detected in some plant species. There is also considerable diversity in the IR size, possibly because the expansion and contraction of the IR regions have been very common events during the evolution of plant species, including those belonging to Fabaceae [16] and Poaceae [17]. The complete cp genome has been used in investigations of phylogenetic relationships, molecular markers, and evolution [18].

Insertions/deletions (indels) and single-nucleotide polymorphisms (SNPs) within the cp genome have been used to rapidly distinguish species [19–21]. Additionally, cp markers have been developed to identify closely related species, including buckwheat and the species of *Solanum*,* Angelica*, and other genera [20–22]. For example, Park et al. [23] used two indel markers (*trnK-trnQ* and *ycf1-ndhF*) to differentiate three* Aconitum* species. Additionally, indels in the *trnL-F*, *trnG-trnS*, and* trnL *introns have been used to analyze the molecular evolution of the *Silene* species cp genome [23]. Thus, indel and SNP cp markers are important for identifying species and investigating molecular evolution.

Several *Aceraceae* species have recently had their cp genome sequences published, including *Acer morrisonense* [24],* Dipteronia sinensis *and *Dipteronia dyeriana* [8], and *Acer griseum* [25]. Chen et al. [26] were the first to report the complete *A. truncatum* cp genome; however, they only focused on the genome composition and phylogenetic relationships. Thus, the *A. truncatum *cp genome was not comprehensively characterized. Compared with the result of Chen et al. [26], in our study, we also found *A. truncatum *is closely related to *A. miaotaiense.* Moreover, we also analyzed the repetition, contraction, and expansion of the IR regions as well as the synonymous and nonsynonymous substitution rates. Highly divergent regions and potential indels were detected via a comparative analysis of six available cp genome sequences. Additionally, on the basis of the results of our comparative analysis of cp genomes, we developed the *ycf4-cemA *indel marker to distinguish six *Acer *species (i.e., *A. buergerianum*, *A. truncatum*, *A. henryi*, *A. negundo*, *A. ginnala*, and *A. tonkinense*). The data presented herein will enrich the genetic information available for the genus* Acer*, provide novel insights into *A. truncatum* evolution, and form an important theoretical basis for increasing the *A. truncatum* seed yield.

## 2. Materials and Methods

### 2.1. DNA Sequencing and Chloroplast Genome Assembly

We collected fresh leaves from *A. truncatum *plants, which were obtained from the *Acer* germplasm collection of the Jiangsu Academy of Agricultural Sciences, Nanjing, Jiangsu, China. The leaves were frozen in liquid nitrogen and stored at −80°C. Total DNA was extracted from the frozen leaves with the DNA Isolation Kit (Aidlab, China). We prepared 350-bp shotgun libraries, which were sequenced according to the double-terminal sequencing method of the Illumina HiSeq X™ Ten platform.

A total of 16.30 GB high-quality clean data (Q30 > 95.23%) were used for assembling the sequence as described by Ferrarini et al. [27]. The cp DNA reads were extracted with SMALT, using the *A. buergerianum *(GenBank accession NC_034744), *A. miaotaiense *(GenBank accession NC_030343), and *A. morrisonense *(GenBank accession KT970611) cp genomes as queries. The reads with 90% similarity were considered to be derived from the cp genome. The data were trimmed with Sickle (https://github.com/najoshi/sickle) (using *q* = 30 as the threshold for trimming and *l* = 50 as the threshold for keeping a read based on length) and assembled with the default parameters of AbySS [28]. Redundant contigs were removed with the CD-Hit program [29] (threshold of 100%) and the unique contigs were merged with the default parameters of Minimus2. The boundary regions of LSC/IRB, IRB/SSC, SSC/IRA, and IRA/LSC of the completed cp genomes were validated with PCR-based sequencing. Details regarding the primers are provided in Supplementary [Supplementary-material supplementary-material-1].

### 2.2. Annotation and Comparative Analysis

The *A. truncatum *cp genome was annotated with DOGMA (http://dogma.ccbb.utexas.edu/). The start and stop codons were coupled manually. All tRNA genes were identified with the default settings of tRNAscan-SE 1.21 [30]. The OGDRAW program was used to visualize the circular *A. truncatum *cp genome map [31]. Codon usage was analyzed with MEGA 6.0 [32]. The cp genomes of six *Acer *species (*A. truncatum*,* A. buergerianum*, *A. davidii*, *A. griseum*, *A. miaotaiense*, and *A. morrisonense*) were compared with mVISTA [33, 34], with the annotated *A. morrisonense *sequence used as the reference.

### 2.3. Analysis of Repeat Structures and Simple Sequence Repeats

Four types of repeat structures (i.e., forward repeat, palindromic repeat, reverse repeat, and complementary repeat) were identified with REPuter [35]. Additionally, tandem repeats were detected with the default settings of the Tandem Repeats Finder program (version 4.07b) [36]. The simple sequence repeats (SSRs) were analyzed with the MISA program. The motif size for mono-, di-, tri-, tetra-, penta-, and hexanucleotide SSRs was set as 10, 5, 4, 3, 3, and 3, respectively [37].

### 2.4. Analysis of Synonymous and Nonsynonymous Substitution Rates

The *A. truncatum*, *Citrus platymamma*,* Dimocarpus longan*, and *Spondias mombin* cp genome sequences were compared to determine the synonymous (*K*_*s*_) and nonsynonymous (*K*_*a*_) substitution rates. The protein-coding exons were separately aligned with MEGA 6.0. The *K*_*s*_ and *K*_*a*_ substitution rates were estimated with DnaSP [38].

### 2.5. Phylogenomic Analyses

A total of 22 whole cp genome sequences of Sapindales species (Supplementary [Supplementary-material supplementary-material-1]) were used for elucidating the evolutionary status of *A. truncatum*, with *Euonymus hamiltonianus *(order Celastrales) serving as the outgroup. The 64 single-copy orthologous genes common among the 23 analyzed genomes were aligned with the default parameters of ClustalW 2.0 [39]. The maximum likelihood (ML) analyses of phylogenetic relationships were completed with RAxML using the GTRGAMMA model [40].

### 2.6. Estimation of the Divergence Time

For the divergence time, we first removed ambiguously aligned sites in the 23 whole genomes data set using GBLOCKS v.0.91b [41] with the flowing parameters: minimum sequences per conserved position, 15; minimum sequences per flank position, 20; maximum number of contiguous nonconserved positions, 8; minimum block length, 10; allowed gap positions, none. Then, the divergence time was estimated with the MCMCTree program of PAML (version 4.9a) [42], with the following parameters: burnin 100000, sampfreq 200, and nsample 10000. Moreover, *E. hamiltonianus *was constrained to be the outgroup, and the root age was constrained by the divergence time of *E. hamiltonianus* from* A. truncatum *(98–117 million years ago) (http://www.timetree.org/).

### 2.7. Development and Validation of the ycf4-cemA Indel Marker

The indel regions were selected based on the results of a similarity search with mVISTA. Additionally, primers were designed with Primer 5. The PCR amplification was performed as described by Ma et al. [43]. To confirm the accuracy of the PCR product sizes, three samples per species were sequenced by the General Biology Company (Nanjing, Jiangsu, China).

## 3. Results and Discussion

### 3.1. Features of the A. truncatum Chloroplast Genome

**Figure 1:**
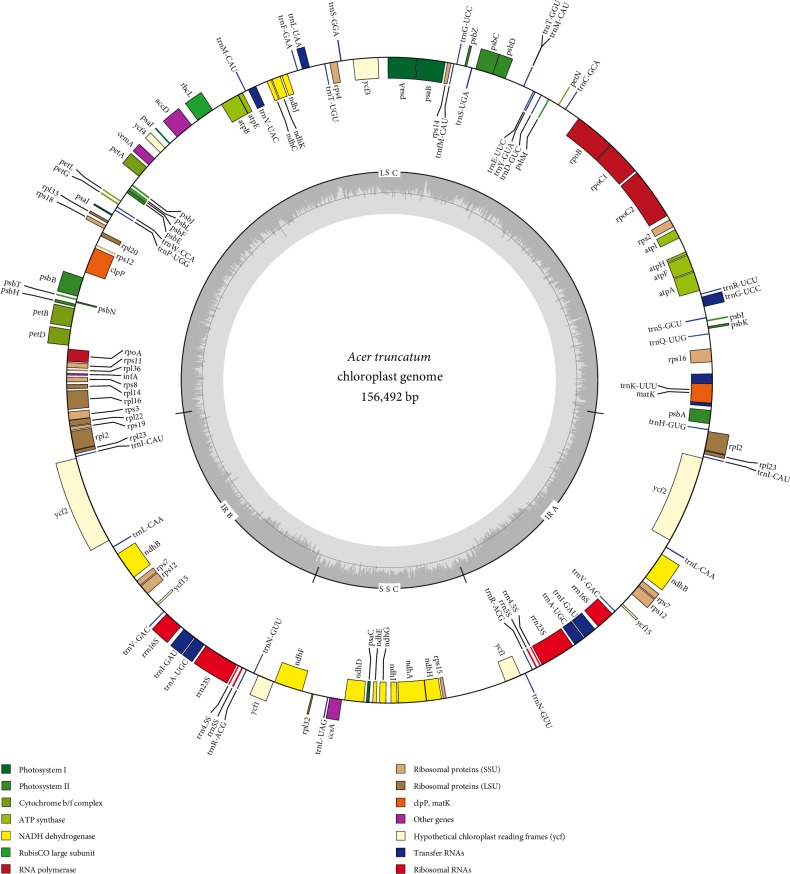
Map of the complete *A. truncatum* chloroplast genome. Genes marked inside the circle are transcribed in a clockwise direction, and those on the outside of the circle are transcribed in a counter-clockwise direction. The dark and light gray areas correspond to the GC and AT contents, respectively.

The *A. truncatum* genome sequence was submitted to the GenBank database (accession number MH638284). Chen et al. [26] was the first to describe the *A. truncatum* cp genomic features. Specifically, they reported that the *A. truncatum* cp genome comprises 156, 262 bp, with an overall GC content of 37.9%. In the current study, we revealed similar structural features, with the *A. truncatum *cp genome consisting of 156, 492 bp and forming a typical quadripartite structure (Figure 1 and Table 1). The LSC region (86, 010 bp) and SSC region (18, 050 bp) were separated by a pair of inverted repeats (IRA and IRB; 26, 216 bp each). The GC content may be an important factor for assessing species similarity. The GC content of the complete *A. truncatum *cp genome was 37.90%, which was the same as the result of Chen et al. [26] and that of the LSC, SSC, and IR regions was 36.10%, 32.10%, and 42.80%, respectively, which is similar to the GC contents reported for other *Acer* species (Table 1) [24, 25]. The rRNA and tRNA genes had the highest GC contents in the IR regions across the complete cp genome, which is a phenomenon that is very common among plant species [44, 45].

We detected 134 genes in the *A. truncatum *cp genome, including 20 duplicated genes in the IR regions, 112 unique functional genes, and 2 pseudogenes. The 112 functional genes comprised 4 rRNA genes, 30 tRNA genes, and 78 protein-coding genes (Table 2). Among the 134 genes in the cp genome, 17 genes contained introns, of which three genes (*ycf3*,* clpP*, and* rps12*) contained two introns and the remaining genes contained one intron (i.e., eight protein-coding genes and six tRNA genes) (Table 2). The *rps12* gene was *trans*-spliced, with its 3′ exon duplicated in the IRs and its 5′ exon located in the LSC region. Interestingly, *trnK-UUU *had the largest intron (2,487 bp) because of the presence of the *matK *gene. The *infA *and* ycf1* genes were designated as pseudogenes. The *infA *gene contained several internal stop codons and the *ycf1* gene was located at the boundary region of IR and SSC (Figure 1).

In this study, we assessed the relative synonymous codon usage (RSCU), which represents the nonuniform synonymous codon usage in coding sequences. Generally, RSCU values >1.00 and <1.00 indicate the codon is used more and less frequently than expected, respectively [46]. The codon usage frequency in the *A. truncatum* cp genome was estimated based on the protein-coding gene sequences (Table 3). The protein-coding genes comprised 77,796 bp encoding 25,932 codons. Leucine and cysteine were the most and least prevalent amino acids encoded by the codons, accounting for 10.82% and 1.17% of the codons, respectively. With the exception of the methionine and tryptophan codons, most of the amino acid codons had sequence biases [e.g., UUA (RSCU = 1.80) for leucine, UCU (RSCU = 1.56) for serine, and UAU (RSCU = 1.60) for tyrosine] (Table 3). Codon usage was generally biased toward A or T (U) with high RSCU values, which is a phenomenon that is very common among the cp genomes of land plant species [47, 48].

### 3.2. Analysis of the Repeats in the A. truncatum Chloroplast Genome

**Figure 2:**
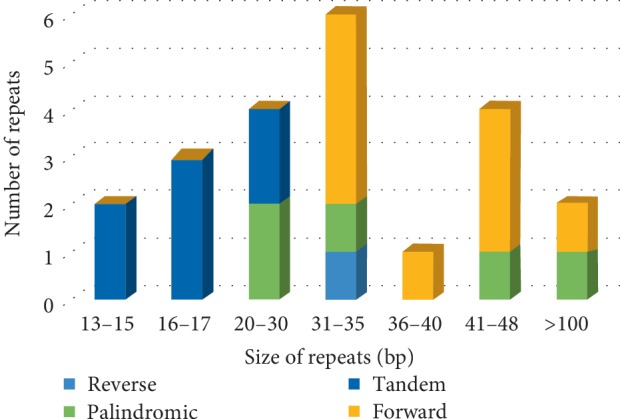
Type and length of repeats in the *A. truncatum* chloroplast genome.

An analysis of the repeats in the* A. truncatum* cp genome revealed 22 long repeats (i.e., one reverse, nine forward, five palindromic, and seven tandem repeats). The only reverse repeat was 35 bp long. The forward and palindromic repeats were mainly longer than 30 bp (Supplementary [Supplementary-material supplementary-material-1] and Figure 2), whereas the tandem repeats were mainly 13–28 bp long (Supplementary [Supplementary-material supplementary-material-1]). Most repeats were located in the intergenic spacers, with the rest located in protein-coding regions and introns. Short dispersed repeats are important for promoting cp genome rearrangements [49].

**Figure 3:**
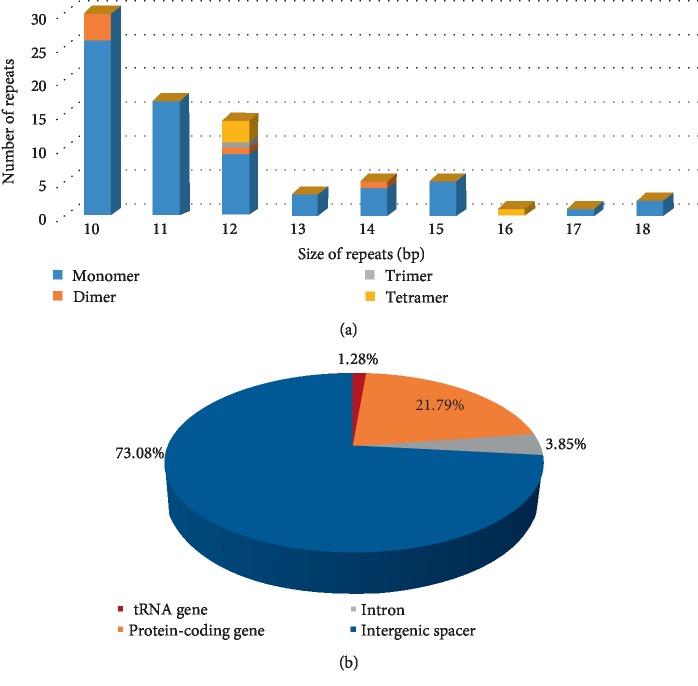
Analysis of SSRs in the *A. truncatum* chloroplast genome. (a) Number of detected SSR types. (b) Frequency of SSRs in the tRNA genes, protein-coding genes, intergenic spacers, and introns.

Simple sequence repeats are useful molecular markers for studying genetic diversity and identifying species [43]. In the current study, we detected 78 perfect microsatellites in the *A. truncatum* cp genome, including 67, 6, 1, and 4 mono-, di-, tri-, and tetranucleotide repeats, respectively; no hexanucleotide repeats were identified (Figure 3(a) and Supplementary [Supplementary-material supplementary-material-1]). Most of these repeats were located in noncoding regions. Additionally, A or T accounted for 94.03% of the mononucleotide repeats, whereas all of the dinucleotide repeats were AT. An examination of the distribution of the SSRs in the *A. truncatum* cp genome indicated that 73.08%, 21.79%, 3.85%, and 1.28% of the SSRs were in the intergenic spacer, protein-coding, intron, and tRNA regions, respectively (Figure 3(b)). Moreover, our data suggest that the *A. truncatum* cp genome contains fewer SSRs than the *A. miaotaiense *cp genome [24]. However, in both of these *Acer *species, the SSRs generally comprise A or T, which contributes to the A/T richness of their cp genomes. These results represent useful information regarding the cp SSR markers that can be applied to investigate the genetic diversity of *A. truncatum *as well as the relationships among species. These markers may also be relevant for selecting germplasms with high nervonic acid contents.

### 3.3. Contraction and Expansion of the IR Regions

**Figure 4:**
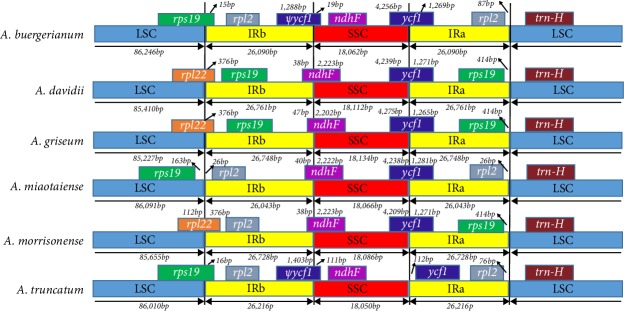
Comparison of the IR boundaries among the chloroplast genomes of six *Acer *species. Boxes above or below the main line indicate the adjacent border genes. ø, pseudogene.

The number and order of genes were highly conserved among the cp genomes of six *Acer* species. However, structural changes were detected in the IR boundaries (Figure 4). These changes represent a common evolutionary event and a major factor influencing the size differences among the cp genomes, implying they have an important evolutionary role in plants [50, 51]. We also compared the boundary regions of IR/LSC and IR/SSC in the cp genomes of* A. buergerianum*,* A. davidii*,* A. griseum*,* A. miaotaiense*,* A. morrisonense*, and *A. truncatum*. In the *A. buergerianum*, *A. miaotaiense*, and *A. truncatum *cp genomes, the *rps19*, *ycf1*, and *rpl2 *genes were detected at the junctions of the LSC/IRb, SSC/IR, and LSC/IRa boundary regions, respectively (Figure 4). However, the *rps19 *gene was located entirely in the LSC region in the *A. miaotaiense *cp genome, but not in the other cp genomes. Additionally, in the *A. buergerianum *and *A. truncatum* cp genomes, the *ycf1 *gene was located in the SSC/IRa border regions, which resulted in a pseudogene in the IRb region. The cp genomes of the other three species (i.e., *A. davidii*,* A. griseum*, and* A. morrisonense*) exhibited a similar trend regarding the IR contraction and expansion. The *rpl22* and *ndhF* genes were located in the LSC/IRb and SSC/IRb regions, respectively. The *rpl22* gene extended 376 bp into the IRb region. In all cp genomes, the *trnH* gene was located in the LSC region. Overall, we detected the contraction and expansion of the IR regions in all six analyzed *Acer* cp genomes.

### 3.4. Comparative Analysis of Six Acer Chloroplast Genomes

**Figure 5:**
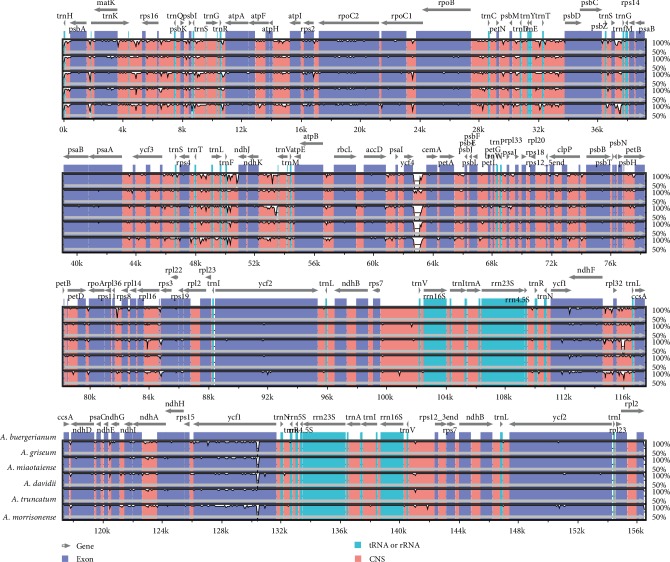
Comparison of the chloroplast genomes of six *Acer* species with mVISTA. The 50% and 100% refer to the similarity among sequences. Gray arrows above the aligned sequences represent genes and their orientation.

A comparative analysis of cp genomes is important for elucidating phylogenetic relationships and identifying species [52, 53]. With the annotated *A. morrisonense* cp genome as the reference, the overall sequence identities among the six analyzed *Acer* cp genomes were determined and visualized with mVISTA (Figure 5). The comparative cp genome analysis proved that the noncoding regions were more diverse than the coding regions, which is consistent with the findings in other plant species [54]. The IR regions were more conserved than the LSC and SSC regions, and four rRNA genes were essentially identical in the six *Acer* species. The intergenic spacers were relatively diverse (e.g., *trnH-psbA*, *matK-rps16*, *petN-psbM*, *petA-psbJ*, and *ycf4-cemA*). The most diverse coding regions were the *matK*, *rps2*, *rpoC2*, *rpoB*, *rps19*, and *ycf1 *sequences. Similar results were observed in previous studies [55, 56]. The highly diverse regions identified in the current study may be relevant for developing markers or genetic barcodes useful for exploring the genetic differentiation among *Aceraceae* species.

### 3.5. Analysis of Synonymous and Nonsynonymous Substitution Rates

**Figure 6:**
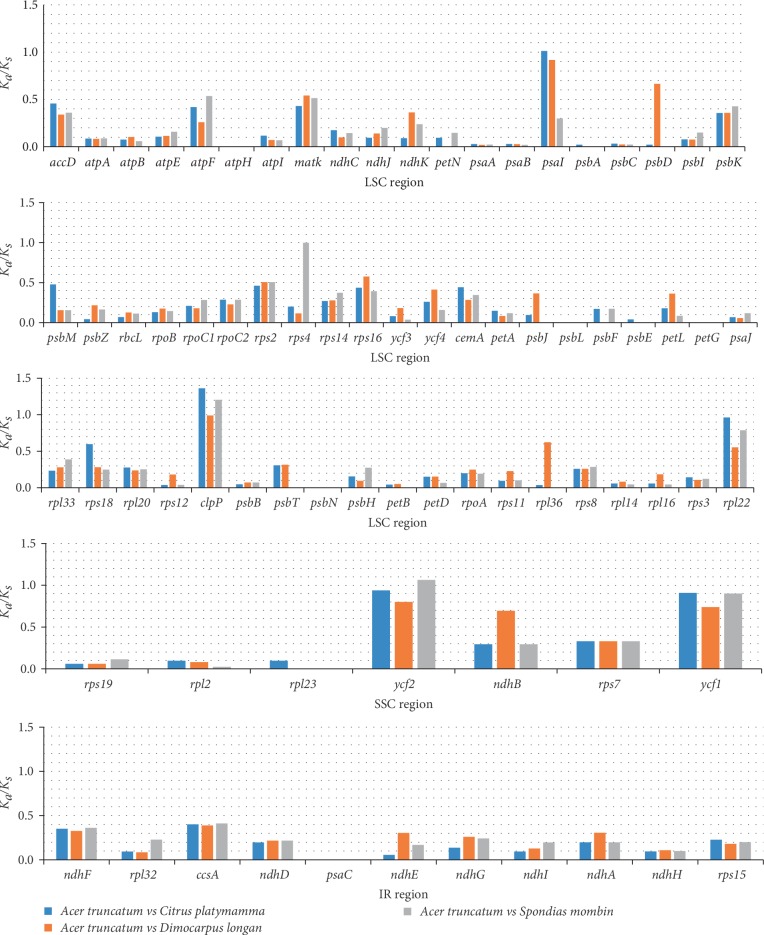
*K*_*a*_/*K*_*s*_ ratios for 78 protein-coding genes in *A. truncatum*, *C. platymamma*, *D. longan*, and *S. mombin*.

In a previous study, the nonsynonymous and synonymous substitution ratio (*K*_*a*_/*K*_*s*_) was used to evaluate the evolutionary forces on some genes [49]. In this study, the *K*_*a*_/*K*_*s*_ ratio was determined for 78 protein-coding genes following the comparison of the *A. truncatum* cp genome with the cp genomes of *C. platymamma*, *D. longan*, and *S. mombin* (Figure 6). Nearly all of the *K*_*a*_/*K*_*s*_ ratios were less than 1.0, implying most of the protein-coding genes were under purifying selection during evolution. However, the *K*_*a*_/*K*_*s*_ ratio of seven genes (*atpF*, *matK*, *psbD*, *rps16*, *rps18*,* rpl36*,* ndhB*, and *ycf1*) was between 0.5 and 1.0. Moreover, the *K*_*a*_/*K*_*s*_ ratio was greater than 1 for *psaIclpP*, *rps4*, *rpl22 *and* ycf2*, which indicated these genes were under positive selection during evolution. High *K*_*a*_/*K*_*s*_ ratios have been reported for some genes, including *ndhC*, *rps16*, and *ycf2 *[49]. These results clearly indicate that cp genes in different plant species may be subjected to diverse selection pressures.

### 3.6. Phylogenetic Analysis

**Figure 7:**
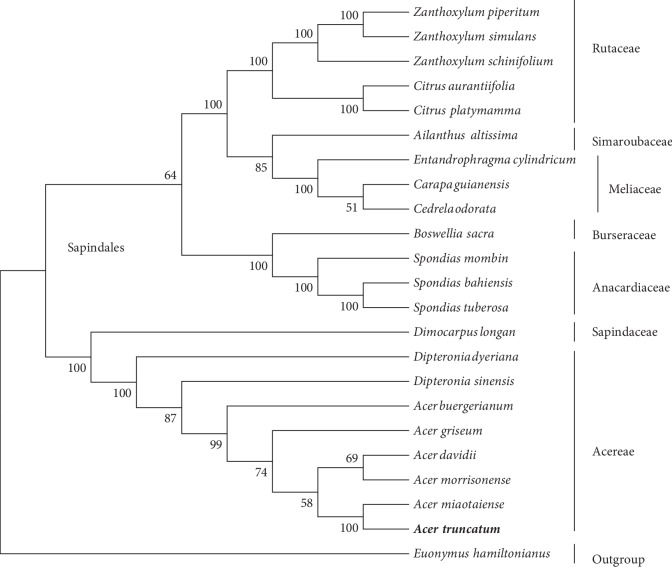
Maximum-likelihood tree based on 64 single-copy orthologous genes shared among 23 species. Numbers at the nodes are bootstrap support values. The position of *A. truncatum* is indicated in bold. *Euonymus hamiltonianus* served as the outgroup.

Chloroplast genome sequences are valuable genomic resources for elucidating evolutionary history and have been widely applied in phylogenetic studies [55–59]. In the current study, to determine the phylogenetic position of *A. truncatum*, 22 complete cp genome sequences of Sapindales species were obtained from the GenBank database (Supplementary [Supplementary-material supplementary-material-1]). A set of 64 single-copy orthologous genes present in the 23 analyzed cp genomes was used to construct phylogenetic trees, with *E. hamiltonianus* serving as the outgroup. All *Aceraceae* species, including *Acer *and *Dipteronia *species, were grouped in one clade, which was consistent with the results of earlier investigations [25, 60, 61]. In a previous study, Chen et al. [25] proved that* A. truncatum* and *A. miaotaiense *are closely related. In our study, we obtain similar phylogenetic topologies, the ML trees also strongly supported the close phylogenetic relationship between *A. truncatum* and *A. miaotaiense *among the *Aceraceae* species, with 100% bootstrap support (Figure 7). Overall, the result of our analysis of cp genomes provides a valuable foundation for future analyses of the phylogenetic affinities of* Acer *species.

### 3.7. Divergence Estimates

**Figure 8:**
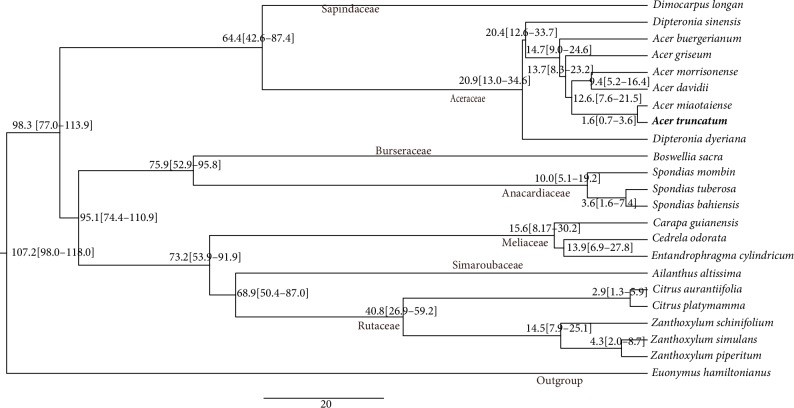
Divergence times of 22 species of Sapindales based on complete chloroplast sequences. Values at nodes indicate divergence dates in millions of years. *Euonymus hamiltonianus* served as the outgroup.

Divergence time estimates were based on a single calibartion point at the root node (107.2 Mya), which is the divergence time of *E. hamiltonianus* from* A. truncatum *(98–117 million years ago) (http://www.timetree.org/). Results of divergence dates for some of the observed clades as well as the upper and lower bounds of the 95% highest posterior density intervals are shown on Figure 8. According to the MCMCTREE time estimates, the estimated divergence date for Burseraceae and Anacardiaceae, Meliaceae, and Simaroubaceae were 75.9 (52.9–95.8) Mya, and 73.2 (53.9–91.9) Mya, respectively. These results are in agreement with recent study [62]. Additionally, the *Spaindaceae* and *Aceraceae* began to split at 64.4 (42.6–87.4) Mya. The divergence time of *Acer *from *Dipteronia* is 14.7 (9.0–24.6) Mya within *Aceraceae* species. Divergence of *A. buergerianum* from a common ancestor with the five other *Aceraceae* species was estimated at 13.7 (8.3–23.2) Mya. Moreover, a recent divergence event between *A. truncatum* and *A. miaotaiense* around 1.6 (0.7–3.6) Mya. These results of our study will provide insights into the evolutionary of *Aceraceae* species.

### 3.8. Development of the ycf4-cemA Indel Marker

**Figure 9:**
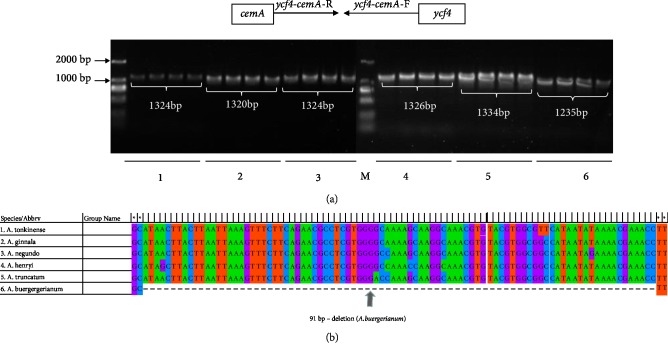
Schematic diagram of the development of the *ycf4-cemA *indel marker in six *Acer *species. (a) Results of the PCR amplification of the *ycf4-cemA *indel marker in the following *Acer *species: (1) *A. tonkinense*, (2) *A. ginnala*, (3) *A. negundo*, (4) *A. henryi*, (5) *A. truncatum*, and (6) *A. buergerianum*; M, 2,000-bp ladder. (b) Alignment of the *ycf4-cemA* marker sequence with MEGA 6.0. The arrow indicates the 91-bp deletion in *A. buergerianum*.

Because indel regions are relatively easy to detect, they are often used to develop markers for identifying species [63]. In the current study, the sequence variability of the large indel regions, which was revealed by sequence alignments with mVISTA, was used to develop markers. A comparison with the *A. truncatum *cp genome sequence detected a 91-bp deletion in the *ycf4-cemA *region of the *A. buergerianum* cp genome. The following six *Acer *species were selected to characterize the *ycf4-cemA* sequence: *A. tonkinense*, *A. ginnala*, *A. negundo*, *A. henryi*, *A. truncatum*, and *A. buergerianum*. To develop indel markers, sequence-specific primers were designed to anneal to the conserved regions flanking *ycf4* and *cemA* (Table 4). The predicted products were successfully amplified with the *ycf4-cemA*-F/R primers for all 24 tested samples (Figure 9(a)). The length of the amplified *ycf4-cemA* sequence was similar for *A. tonkinense*, *A. ginnala*, *A. negundo*, *A. henryi*, *A. truncatum*, and *A. buergerianum*. In contrast, the corresponding sequence in *A. buergerianum *was shorter because of the 91-bp deletion (Figures 9(a) and 9(b)). As presented in Figure 9(a).* A. tonkinense*, *A. ginnala*, *A. negundo*, *A. henryi*, *A. truncatum*, and *A. buergerianum *yielded amplicons of 1,324, 1,320, 1,324, 1,326, 1,334, and 1,235 bp, respectively. Two poly-thymine repeats were identified in the sequenced fragments. Interestingly,* A. truncatum* had an 8-bp insertion that was lacking in the other species. Other deletions are listed in Supplementary [Supplementary-material supplementary-material-1]. The predicted sizes of the indels were consistent with the sizes of the fragments amplified from the 24 samples analyzed in this study. Indel markers have commonly been used to distinguish closely related species in previous studies [22, 23]. However, *Acer *species have not been identified using this approach. Thus, indel markers may represent an important resource for identifying species. The *ycf4-cemA* indel marker developed in this study may be applicable for species classifications and the identification of *Acer* species.

## Figures and Tables

**Table 1:** Details regarding the *A. truncatum* chloroplast genome.

Characteristics		Chloroplast features	
Total cp DNA size (bp)		156, 492	
LSC size (bp)		86, 010	
SSC size (bp)		18, 050	
IR size (bp)		26, 216	
Total GC content (%)		37.9	
GC content of LSC (%)		36.1	
GC content of SSC (%)		32.2	
GC content of IR (%)		42.8	
Total CDS length (bp)		77, 796	
Protein-coding genes		78	
tRNAs		30	
rRNAs		4	
Genes duplicated		20	
Genes with a single intron(s)		15	
Gene with two introns		3	
Pseudogenes		2	

**Table 2:** Gene composition of the *A. truncatum* chloroplast genome.

	Classification of genes	Gene name	Number
1	Photosystem I	psaA, B, C, I, J	5
2	ATP synthase	atpA, B, E, F^a^, H, I	6
3	Photosystem II	psbA, B, C, D, E, F, H, I, J, K, M, N, T, Z	15
4	Rubisco	rbcL	1
5	Cytochrome	petA, B^a^, D^a^, G, L, N	6
6	NADH oxidoreductase	ndhA^a^, B^a,c^(×2), C, D, E, F, G, H, I, J, K	12
7	Ribosomal proteins (SSU)	rps2, 3, 4, 7^c^(×2), 8, 11, 12(×2)^a,c,d^, 14, 15, 16^a^, 18, 19^c^	14
8	Ribosomal proteins (LSU)	rpl2^a,c^(×2), 14, 16^a^, 20, 22, 23^c^(×2), 32, 33, 36	11
9	RNA polymerase	rpoA, rpoB, rpoC1^a^, rpoC2	4
10	Ribosomal RNAs	rrn4.5^c^(×2), 5^c^(×2), 16^c^(×2), 23^c^(×2)	8
11	Other proteins	accD, ccsA, matK, clpP^b^, cemA	5
12	Transfer RNAs	trnA-UGC(×2)^a,c^, trnC-GCA, trnD-GUC, trnE-UUC, trnF-GAA, trnfM-CAU, trnG-UCC^a^, trnG-GCC, trnH-GUG(×2), trnI-GAU (×2)^a,c^, trnL-CAA^c^(×2), trnL-UAA^a^, trnL-UAG, trnM-CAU, trnN-GUU(×2)^c^, trnP-UGG, trnQ-UUG, trnK-UUU^a^ trnR-UCU, trnS-GCU, trnS-GGA, trnS-UGA, trnT-GGU, trnT-UGU, trnV-GAC(×2)^c^, trnR-ACG (×2)^c^, trnV-UAC^a^, trnW-CCA, trnY-GUA, trnI-CAU(×2)^ c^	38
13	Hypothetical proteins	ycf1, 2^c^(×2), 3^b^, 4, 15^c^(×2),	7
14	Pseudogenes	infA, ycf1	2
Total			134

^a^A single intron gene.

^b^Two introns gene.

^c^Two gene copies in IRs regions.

^d^Gene divided into two independent transcription units.

^e^Pseudogene.

**Table 3:** Codon usage in the *A. truncatum *chloroplast genome.

Amino acid	Codon	No.	RSCU^a^	Amino acid	Codon	No.	RSCU
Phe	UUC	555	0.74	Tyr	UAC	187	0.4
Phe	UUU	955	1.26	Tyr	UAU	759	1.6
Leu	CUC	227	0.5	His	CAC	161	0.5
Leu	UUG	555	1.21	Stop	UAG^∗^	24	0.73
Leu	UUA	822	1.80	Stop	UAA^∗^	53	1.62
Leu	CUU	560	1.22	His	CAU	479	1.5
Leu	CUG	207	0.45	Gln	CAG	224	0.5
Leu	CUA	376	0.82	Gln	CAA	667	1.5
Ile	AUC	445	0.62	Asn	AAC	293	0.48
Ile	AUU	1054	1.46	Asn	AAU	921	1.52
Ile	AUA	668	0.92	Lys	AAA	944	1.46
Met	AUG	615	1	Lys	AAG	345	0.54
Val	GUU	522	1.45	Asp	GAU	809	1.56
Val	GUC	182	0.51	Asp	GAC	229	0.44
Val	GUG	205	0.57	Glu	GAG	362	0.55
Val	GUA	532	1.48	Glu	GAA	966	1.45
Ser	UCC	352	1.03	Cys	UGC	81	0.53
Ser	UCU	531	1.56	Cys	UGU	222	1.47
Ser	UCG	202	0.59	Trp	UGG	440	1
Ser	UCA	433	1.27	Stop	UGA^∗^	21	0.64
Pro	CCC	221	0.8	Arg	CGC	126	0.49
Pro	CCU	417	1.52	Arg	CGU	308	1.19
Pro	CCG	153	0.56	Arg	CGG	144	0.56
Pro	CCA	309	1.12	Arg	CGA	352	1.36
Thr	ACC	256	0.78	Ser	AGC	132	0.39
Thr	ACU	501	1.53	Ser	AGU	397	1.16
Thr	ACG	160	0.49	Arg	AGG	182	0.7
Thr	ACA	390	1.19	Arg	AGA	441	1.7
Ala	GCC	236	0.66	Gly	GGC	188	0.41
Ala	GCU	617	1.74	Gly	GGU	591	1.29
Ala	GCG	194	0.55	Gly	GGG	338	0.74
Ala	GCA	373	1.05	Gly	GGA	721	1.57

^a^ Relative synonymous codon usage.

^∗^ Stop codon.

**Table 4:** Details regarding the primers used to develop the *ycf4-cemA *marker.

Primer name	Primer sequence (5′ > 3′)	Position
*ycf4-cemA*-F	GCTGGGCGTTTATCCTTTTT	*ycf4-cemA*
*ycf4-cemA*-R	GGATTGTTTCTTTGTGGAGC	

## Data Availability

The *Acer truncatum* chloroplast genome sequence was deposited in the GenBank database (accession MH638284).

## Supplementary Materials

Supplementary Materials Supplementary Table S1: PCR-based sequence validation of junctions between the large single-copy (LSC), small single-copy (SSC), and inverted repeat (IRa and IRb) regions of the *A. truncatum* chloroplast genome. Supplementary Table S2: Long repeat sequences in the *A. truncatum* chloroplast genome. Supplementary Table S3: Details regarding the tandem repeats in the *A. truncatum* chloroplast genome. Supplementary Table S4: Distribution of SSRs in the *A. truncatum* chloroplast genome. Supplementary Table S5: Details regarding the chloroplast genome sequences used for the phylogenetic analysis. Supplementary Table S6: The sequences variability of sequenced fragments.

## References

[1] Guo X., Wang R., Chang R. (2014). Effects of nitrogen addition on growth and photosynthetic characteristics of *Acer truncatum* seedlings. *Dendrobiology*.

[2] Tang W., Wang J., Xu J., Wang L., Huang J., Chen Y. (2012). Advances of chemical composition of medicinal plants in *Aceraceae*. *Northern Horticulture*.

[3] Wang X.-Y., Fan J.-S., Wang S.-Y., Sun R.-C. (2006). A new resource of nervonic acid from purpleblow maple (*Acer truncatum*) seed oil. *Forest Products Journal*.

[4] Yang R.-N., Zhang L.-X., Li P.-W. (2018). A review of chemical composition and nutritional properties of minor vegetable oils in china. *Trends in Food Science & Technology*.

[5] Daniell H., Lin C.-S., Yu M., Chang W.-J. (2016). Chloroplast genomes: diversity, evolution, and applications in genetic engineering. *Genome Biology*.

[6] Tetlow I. J., Rawsthorne S., Raines C., Emes M. J., Moller S. G. (2009). Plastid metabolic pathways. *In Annual Plant Reviews, Plastids*.

[7] Ohyama K., Fukuzawa H., Kohchi T. (1986). Chloroplast gene organization deduced from complete sequence of liverwort* Marchantia polymorpha *chloroplast DNA. *Nature*.

[8] Green B. R. (2011). Chloroplast genomes of photosynthetic eukaryotes. *Plant Journal*.

[9] Zhou T., Chen C., Wei Y. (2016). Comparative transcriptome and chloroplast genome analyses of two related *Dipteronia* species. *Frontiers in Plant Science*.

[10] Liu H.-Y., Yu Y., Deng Y.-Q., Li J., Huang Z.-X., Zhou S.-D. (2018). The chloroplast genome of *Lilium henrici*: genome structure and comparative analysis. *Molecules*.

[11] Zheng X.-M., Wang J., Feng L. (2017). Inferring the evolutionary mechanism of the chloroplast genome size by comparing whole-chloroplast genome sequences in seed plants. *Scientific Reports*.

[12] Ogihara Y., Terachi T., Sasakuma T. (1988). Intramolecular recombination of chloroplast genome mediated by short direct-repeat sequences in wheat species. *Proceedings of the National Academy of Sciences of the United States of America*.

[13] Wang W., Chen S., Zhang X. (2018). Whole-genome comparison reveals heterogeneous divergence and mutation hotspots in chloroplast genome of *Eucommia ulmoides* oliver. *International Journal of Molecular Sciences*.

[14] Gu C., Tembrock L., Zheng S., Wu Z. (2018). The complete chloroplast genome of *Catha edulis*: a comparative analysis of genome features with related species. *International Journal of Molecular Sciences*.

[15] Wang W.-C., Chen S.-Y., Zhang X.-Z. (2016). Chloroplast genome evolution in actinidiaceae: *clpP* loss, heterogenous divergence and phylogenomic practice. *PLoS One*.

[16] Wicke S., Schneeweiss G. M., Müller K. F., Quandt D. (2011). The evolution of the plastid chromosome in land plants: gene content, gene order, gene function. *Plant Molecular Biology*.

[17] Ma P.-F., Zhang Y.-X., Zeng C.-X., Guo Z.-H., Li D.-Z. (2014). Chloroplast phylogenomic analyses resolve deep-level relationships of an intractable bamboo tribe *Arundinarieae* (Poaceae). *Systematic Biology*.

[18] Parks M., Cronn R., Liston A. (2009). Increasing phylogenetic resolution at low taxonomic levels using massively parallel sequencing of chloroplast genomes. *BMC Biology*.

[19] Kim K., Lee S. C., Lee J. (2015). Comprehensive survey of genetic diversity in chloroplast genomes and 45S nrDNAs within *Panax ginseng* species. *PLoS One*.

[20] Cho K.-S., Yun B.-K., Yoon Y.-H. (2015). Complete Chloroplast genome sequence of tartary buckwheat (*Fagopyrum tataricum*) and comparative analysis with common buckwheat (*F. esculentum*). *PLoS One*.

[21] Cho K. S., Cheon K. S., Hong S. Y. (2016). Complete chloroplast genome sequences of *Solanum commersonii* and its application to chloroplast genotype in somatic hybrids with *Solanum tuberosum*. *Plant Cell Reports*.

[22] Park I., Yang S., Kim W. J. (2019). Sequencing and comparative analysis of the chloroplast genome of *Angelica polymorpha* and the development of a novel Indel marker for species identification. *Molecules*.

[23] Ingvarsson P. K., Ribstein S., Taylor D. R. (2003). Molecular evolution of insertions and deletion in the chloroplast genome of* silene*. *Molecular Biology and Evolution*.

[24] Li Z.-H., Xie Y.-S., Zhou T., Jia Y., He Y. L., Yang J. (2015). The complete chloroplast genome sequence of *Acer morrisonense* (*Aceraceae*). *Mitochondrial DNA Part A*.

[25] Wang W. C., Chen S.-Y., Zhang X.-Z. (2017). The complete chloroplast genome of the endangered Chinese paperbark maple, *Acer griseum*, (*Sapindaceae*). *Conservation Genetics Resources*.

[26] Chen S., Liu B., Zhang S., Huang J. (2019). The complete chloroplast genome of *Acer truncatum* bunge (*Aceraceae*). *Mitochondrial DNA Part B*.

[27] Ferrarini M., Moretto M., Ward J. A. (2013). An evaluation of the PacBio RS platform for sequencing and *de novo* assembly of a chloroplast genome. *BMC Genomics*.

[28] Simpson J. T., Wong K., Jackman S. D. (2009). ABySS: a parallel assembler for short read sequence data. *Genome Research*.

[29] Li W., Godzik A. (2006). Cd-hit: a fast program for clustering and comparing large sets of protein or nucleotide sequences. *Bioinformatics*.

[30] Liu C., Shi L., Zhu Y. (2012). CpGAVAS, an integrated web server for the annotation, visualization, analysis, and GenBank submission of completely sequenced chloroplast genome sequences. *BMC Genomics*.

[31] Lohse M., Drechsel O., Bock R. (2007). OrganellarGenomeDRAW (OGDRAW): a tool for the easy generation of high-quality custom graphical maps of plastid and mitochondrial genomes. *Current Genetics*.

[32] Tamura K., Stecher G., Peterson D., Filipski A., Kumar S. (2013). MEGA6: molecular evolutionary genetics analysis version. *Molecular Biology Evolution*.

[33] Frazer K. A., Pachter L., Poliakov A., Rubin E. M., Dubchak I. (2004). VISTA: computational tools for comparative genomics. *Nucleic Acids Research*.

[34] Mayor C., Brudno M., Schwartz J. R. (2000). VISTA: visualizing global DNA sequence alignments of arbitrary length. *Bioinformatics*.

[35] Kurtz S., Choudhuri J. V., Ohlebusch E., Schleiermacher C., Stoye J., Giegerich R. (2001). The manifold applications of repeat analysis on a genomic scale. *Nucleic Acids Research*.

[36] Benson G. (1999). Tandem repeats finder: a program to analyze DNA sequences. *Nucleic Acids Research*.

[37] Thiel T. (2003). MISA-Microsatellite Identification Tool. http://pgrc.ipk-gatersleben.de/misa/..

[38] Librado P., Rozas J. (2009). DnaSP v5: a software for comprehensive analysis of DNA polymorphism data. *Bioinformatics*.

[39] Larkin M. A., Blackshields G., Brown N. P. (2007). Clustal W and Clustal X version 2.0. *Bioinformatics*.

[40] Stamatakis A. (2014). RAxML version 8: a tool for phylogenetic analysis and post-analysis of large phylogenies. *Bioinformatics*.

[41] Talavera G., Castresana J. (2007). Improvement of phylogenies after removing divergent and ambiguously aligned blocks from protein sequence alignments. *Systematic Biology*.

[42] Yang Z. (2007). PAML 4: phylogenetic analysis by maximum likeli-hood. *Molecular Biology and Evolution*.

[43] Ma Q. Y., Li S. X., Bi C. W., Hao Z., Sun C., Ye N. (2017). Complete chloroplast genome sequence of a major economic species, *Ziziphus jujuba* (*Rhamnaceae*). *Current Genetics*.

[44] Shen X., Wu M., Liao B. (2017). Complete chloroplast genome sequence and phylogenetic analysis of the medicinal plant *Artemisia annua*. *Molecules*.

[45] He Y., Xiao H., Deng C., Xiong L., Yang J., Peng C. (2016). The complete chloroplast genome sequences of the medicinal plant *Pogostemon cablin*. *International Journal of Molecular Sciences*.

[46] Sharp P. M., Li W. H. (1987). The codon adaptation Index-a measure of directional synonymous codon usage bias, and its potential applications. *Nucleic Acids Research*.

[47] Kim K. J., Lee H. L. (2004). Complete chloroplast genome sequences from Korean ginseng (*Panax schinseng* Nees) and comparative analysis of sequence evolution among 17 vascular plants. *DNA Research*.

[48] Zhou J., Cui Y., Chen X (2018). Complete chloroplast genomes of *Papaver rhoeas* and *Papaver orientale*: molecular structures, comparative analysis, and phylogenetic analysis. *Molecules*.

[49] Wu M., Li Q., Hu Z., Li X., Chen S. (2017). The complete *Amomum kravanh* chloroplast genome sequence and phylogenetic analysis of the commelinids. *Molecules*.

[50] Raubeson L. A., Peery R., Chumley T. W. (2007). Comparative chloroplast genomics: analyses including new sequences from the angiosperms *Nuphar advena* and *Ranunculus macranthus*. *BMC Genomics*.

[51] Wang W., Chen S., Zhang X. (2018). Whole-genome comparison reveals divergent IR borders and mutation hotspots in chloroplast genomes of herbaceous bamboos (Bambusoideae:*Olyreae*). *Molecules*.

[52] Chen S., Xu J., Liu C. (2012). Genome sequence of the model medicinal mushroom *Ganoderma lucidum*. *Nature Communication*.

[53] Zhihai H., Jiang X., Shuiming X. (2016). Comparative optical genome analysis of two pangolin species: *Manis pentadactyla* and *Manis javanica*. *GigaScience*.

[54] Chi X., Wang J., Gao Q., Zhang F., Chen S. (2018). The complete chloroplast genomes of two *Lancea* species with comparative analysis. *Molecules*.

[55] Zeng S., Zhou T., Han K., Yang Y., Zhao J., Liu Z.-L. (2017). The complete chloroplast genome sequences of six *Rehmannia* species. *Genes*.

[56] Hong S. Y., Cheon K. S., Yoo K. O. (2017). Complete chloroplast genome sequences and comparative analysis of *Chenopodium quinoa* and *C. album*. *Frontiers in Plant Science*.

[57] Gitzendanner M. A., Soltis P. S., Wong G. K., Ruhfel B. R., Soltis D. E. (2018). Plastid phylogenomic analysis of green plants: a billion years of evolutionary history. *American Journal of Botany*.

[58] Du Y., Bi Y., Chen X., Yang F., Xue J., Zhang X. (2016). The complete chloroplast genome of *Lilium cernuum*: genome structure and evolution. *Conservation Genetics Resources*.

[59] Cao J.-L., Jiang D., Zhao Z.-Y. (2018). Development of chloroplast genomic resources in Chinese Yam, (*Dioscorea polystachya*). *BioMed Research International*.

[60] Zhou T., Chen C., Wei Y. (2016). Comparative transcriptome and chloroplast genome analyses of two related *Dipteronia* species. *Frontiers in Plant Science*.

[61] Zhao J., Xu Y., Xi L., Yang J., Chen H., Zhang J. (2018). Characterization of the chloroplast genome sequence of *Acer miaotaiense*: comparative and phylogenetic analyses. *Molecules*.

[62] Muellner-Riehl A. N., Weeks A., Clayton J. W., Buerki S. (2016). Molecular phylogenetics and molecular clock dating of sapindales based on plastid *rbcl*, *atpb* and *trnl-trnf* DNA sequences. *Taxon*.

[63] Suo Z., Jia Z., Lu Q. (2012). Distinguishing *Haloxylon persicum* and *H. Ammodendron* (*Haloxylon* Bunge, *Amaranthaceae*) using DNA marker. *AASRI Procedia*.

