# Supplementary material for: Characterization of the Complete Chloroplast Genome of Acer truncatum Bunge (Sapindales: Aceraceae): A New Woody Oil Tree Species Producing Nervonic Acid
Source: Biomed Res Int. 2019 Nov 24;2019:7417239. doi: 10.1155/2019/7417239 (PMC6925723; doi:10.1155/2019/7417239)
Supplement: Supplementary Materials — Supplementary Table S1: PCR-based sequence validation of junctions between the large single-copy (LSC), small single-copy (SSC), and inverted repeat (IRa and IRb) regions of the A. truncatum chloroplast genome. Supplementary Table S2: Long repeat sequences in the A. truncatum chloroplast genome. Supplementary Table S3: Details regarding the tandem repeats in the A. truncatum chloroplast genome. Supplementary Table S4: Distribution of SSRs in the A. truncatum chloroplast genome. Supplementary Table S5: Details regarding the chloroplast genome sequences used for the phylogenetic analysis. Supplementary Table S6: The sequences variability of sequenced fragments. [file 7417239.f1.docx]

**Table S1.** PCR-based sequence validation of junctions between the large single-copy (LSC), small single-copy (SSC), and inverted repeat (IRa and IRb) regions of the *A. truncatum* chloroplast genome.

| Primer Name | Primer Sequence  (5'>3') | Start position (bp) | End position (bp) | Identify (%) |
| --- | --- | --- | --- | --- |
| LSC/IRB-F | GTTTAGTTTTCGGTTCGCAC | 84, 918 | 86, 253 | 100 |
| LSC/IRB-R | TAAGCGTCCTGTAGTAAGAG |  |  |  |
| IRB/SSC-F | TCCCCTACAAAAAAACTGGT | 112, 327 | 112, 993 | 100 |
| IRB/SSC-R | CTATGGGGTAAAGAAGAGCC |  |  |  |
| SSC/IRA-F | AGTTTCGCCCCTGTTTTTCT | 129, 772 | 130, 833 | 100 |
| SSC/IRA-R | GACTAAACAGGAACAAGAGG |  |  |  |
| IRA/SSC-F | GGGAAGGGGTTTTGATTGAT | 155, 961 | 428 | 100 |
| IRA/SSC-R | ATCCTCTTCCAATAAATCGG |  |  |  |

**Table S2.** Long repeat sequences in the *A. truncatum* chloroplast genome.

| **No.** | **Size (bp)** | **Type** | **Start 1**  **(bp)** | **Location 1** | **Start2**  **(bp)** | **Location 2** | **Region** | **E-Value** |
| --- | --- | --- | --- | --- | --- | --- | --- | --- |
| 1 | 121 | F | 130, 280 | *IGS* | 130, 399 | *IGS* | IRA | 9.75×10^-64^ |
| 2 | 48 | F | 48, 401 | *IGS* | 48, 441 | *IGS* | LSC | 1.25×10^-17^ |
| 3 | 41 | F | 40, 023 | *psaB(CDS)* | 42, 247 | *psaB(CDS)* | LSC | 1.05×10^-11^ |
| 4 | 41 | F | 100, 187 | *IGS* | 122, 505 | *ndhA(CDS)* | IRB,SSC | 1.05×10^-11^ |
| 5 | 39 | F | 45, 029 | *ycf3(intron)* | 100, 189 | *IGS* | LSC,IRB | 5.62×10^-9^ |
| 6 | 33 | F | 45, 035 | *ycf3(intron)* | 122, 513 | *ndhA(CDS)* | LSC,SSC | 9.24×10^-9^ |
| 7 | 31 | F | 77, 038 | *petB(intron)* | 77, 066 | *petB(intron)* | LSC | 1.39×10^-7^ |
| 8 | 31 | F | 151, 652 | *ycf2(CDS)* | 151, 673 | *ycf2(CDS)* | IRA | 6.25×10^-6^ |
| 9 | 31 | F | 128, 462 | *IGS* | 129, 313 | *IGS* | SSC | 1.81×10^-4^ |
| 10 | 35 | R | 9, 175 | *IGS* | 9, 185 | *IGS* | LSC | 1.03×10^-6^ |
| 11 | 41 | P | 122, 505 | *ndhA(intron)* | 142, 274 | *IGS* | SSC,IRA | 1.05×10^-11^ |
| 12 | 30 | P | 8, 689 | *IGS* | 46, 775 | *trnS-GGA* | LSC | 5.97×10^-9^ |
| 13 | 31 | P | 90, 798 | *ycf2(CDS)* | 151, 652 | *ycf2(CDS)* | IRB,IRA | 6.25×10^-6^ |
| 14 | 30 | P | 36, 537 | *IGS* | 46, 775 | *trnS-GGA* | LSC | 6.55×10^-4^ |
| 15 | 121 | P | 111, 982 | *ycf1(CDS)* | 130, 280 | *rps15-ycf1* | SSC | 9.75×10^-64^ |

Note: IGS, intergenic spacer.

**Table S3.** Details regarding the tandem repeats in the *A. truncatum* chloroplast genome.

| **Number** | **Size(bp)** | **Repeat unit** | **start** | **end** | **Location** |
| --- | --- | --- | --- | --- | --- |
| 1 | 15 | TTCGATATGAAAGGA(×2) | 6, 429 | 6, 458 | *IGS(rps16,* *trnQ-UUG)* |
| 2 | 17 | GATAAGAAAAAAATGAA(×2) | 48, 801 | 48, 834 | *IGS(rps4, trnL-UAA )* |
| 3 | 20 | AATATCTATTCTACAAATTC(×2) | 50, 248 | 50, 288 | *IGS(trnF-GAA , ndhJ)* |
| 4 | 28 | ACAAATTAAAATAACAGATGAAAGAAAA(×2) | 77, 041 | 77, 097 | *petB(intron)* |
| 5 | 16 | GTAAAATCCAGTAGAA(×2) | 78, 398 | 78, 429 | *petD (intron)* |
| 6 | 17 | CTTTTTTAACTTGATTC(×2) | 79, 438 | 79, 472 | *IGS( petD , rpoA )* |
| 7 | 13 | AAAAGAATAAAAG(×2) | 84, 493 | 84, 518 | *IGS( rpl16, rps3)* |

Note: *IGS*, intergenic spacer.

**Table S4.** Distribution of SSRs in the *A. truncatum* chloroplast genome.

| **Type** | **Motif** | **Size (bp)** | **Start** | **End** | **Region** | **Location** |
| --- | --- | --- | --- | --- | --- | --- |
| **Monomer** | A | 10 | 6, 257 | 6, 266 | LSC | *IGS(matK,rps16)* |
|  | A | 11 | 8, 099 | 8, 109 | LSC | *IGS(psbK,psbI)* |
|  | T | 10 | 8, 228 | 8, 237 | LSC | *IGS(psbK,psbI)* |
|  | C | 11 | 8, 399 | 8, 409 | LSC | *psbI* |
|  | A | 15 | 8, 785 | 8, 799 | LSC | *IGS(trnS-GCU,trnG-UCC)* |
|  | T | 10 | 9, 060 | 9, 069 | LSC | *IGS(trnS-GCU,trnG-UCC)* |
|  | T | 10 | 12, 413 | 12, 422 | LSC | *IGS(atpA,atpF)* |
|  | T | 14 | 13, 004 | 13, 017 | LSC | *atpF(intron)* |
|  | T | 10 | 14, 858 | 14, 867 | LSC | *IGS(atpF,atpI)* |
|  | A | 14 | 15, 200 | 15, 213 | LSC | *IGS(atpF,atpI)* |
|  | T | 14 | 19, 133 | 19, 146 | LSC | *rpoC1* |
|  | T | 11 | 23, 540 | 23, 550 | LSC | *rpoC2(intron)* |
|  | T | 10 | 26, 784 | 26, 793 | LSC | *rpoB* |
|  | T | 11 | 28, 273 | 28, 283 | LSC | *IGS(rpoB,trnC-GCA)* |
|  | T | 12 | 30, 218 | 30, 229 | LSC | *IGS(psbM,trnD-GUC)* |
|  | T | 15 | 31, 887 | 31, 901 | LSC | *IGS(trnE-UUC,trnT-GGU)* |
|  | A | 10 | 37, 959 | 37, 968 | LSC | *IGS(trnG-UCC,trnfM-CAU)* |
|  | A | 12 | 43, 645 | 43, 656 | LSC | *IGS(psaA,ycf3)* |
|  | T | 11 | 44, 696 | 44, 706 | LSC | *ycf3(intron)* |
|  | A | 10 | 46, 540 | 46, 549 | LSC | *IGS(ycf3,trnS-GGA)* |
|  | T | 14 | 48, 522 | 48, 535 | LSC | *IGS(trnT-UGU,trnL-UAA)* |
|  | A | 12 | 48, 967 | 48, 978 | LSC | *IGS(trnT-UGU,trnL-UAA)* |
|  | T | 10 | 49, 932 | 49, 941 | LSC | *IGS(trnL-UAA,trnF-GAA)* |
|  | T | 11 | 52, 308 | 52, 318 | LSC | *IGS(ndhK,ndhC)* |
|  | A | 11 | 52, 991 | 53, 001 | LSC | *IGS(ndhC,trnV-UAC)* |
|  | A | 15 | 53, 407 | 53, 421 | LSC | *IGS(ndhC,trnV-UAC)* |
|  | T | 12 | 53, 723 | 53, 734 | LSC | *trnV-UAC* |
|  | A | 10 | 61, 689 | 61, 698 | LSC | *psaI* |
|  | A | 10 | 61, 878 | 61, 887 | LSC | *IGS(psaI,ycf4)* |
|  | T | 11 | 62, 861 | 62, 871 | LSC | *IGS(ycf4,cemA)* |
|  | A | 13 | 657, 16 | 65, 728 | LSC | *IGS(petA,psbJ)* |
|  | A | 10 | 66, 359 | 66, 368 | LSC | *psbF* |
|  | T | 11 | 66, 999 | 67, 009 | LSC | *IGS(psbE,petL)* |
|  | T | 10 | 69, 634 | 69, 643 | LSC | *IGS(rpl33,rps18)* |
|  | T | 12 | 72, 148 | 72, 159 | LSC | *IGS(rps12,clpP)* |
|  | A | 11 | 72, 804 | 72, 814 | LSC | *IGS(clpP,psbB)* |
|  | T | 12 | 73, 134 | 73, 145 | LSC | *IGS(clpP,psbB)* |
|  | A | 11 | 77, 186 | 77, 196 | LSC | *petB* |
|  | A | 10 | 78, 605 | 78, 614 | LSC | *petB* |
|  | C | 12 | 81, 555 | 81, 566 | LSC | *IGS(rpl36,infA)* |
|  | A | 13 | 82, 868 | 82, 880 | LSC | *IGS(rpl14,rpl16)* |
|  | T | 12 | 84, 465 | 84, 476 | LSC | *IGS(rpl16,rps3)* |
|  | T | 10 | 86, 003 | 86, 012 | LSC,IRB | *rps19* |
|  | C | 10 | 99, 518 | 99, 527 | IRB | *IGS(rps19,ycf15)* |
|  | A | 18 | 109, 579 | 109, 596 | IRB | *IGS(rrn5S,trnR-ACG)* |
|  | T | 10 | 111, 912 | 111, 921 | IRB | *ycf1* |
|  | A | 10 | 112, 075 | 112, 084 | IRB | *ycf1* |
|  | A | 10 | 112, 194 | 112, 203 | IRB | *IGS(ycf1,ndhF)* |
|  | A | 15 | 115, 110 | 115, 124 | SSC | *IGS(ndhF,rpl32)* |
|  | A | 17 | 115, 561 | 115, 577 | SSC | *IGS(rpl32,trnL-UAG)* |
|  | T | 13 | 115, 712 | 115, 724 | SSC | *IGS(rpl32,trnL-UAG)* |
|  | T | 10 | 115, 829 | 115, 838 | SSC | *IGS(rpl32,trnL-UAG)* |
|  | T | 12 | 119, 345 | 119, 356 | SSC | *IGS(ndhD,psaC)* |
|  | A | 10 | 122, 803 | 122, 812 | SSC | *ndhA(intron)* |
|  | T | 15 | 122, 917 | 122, 931 | SSC | *ndhA(intron)* |
|  | T | 10 | 123, 203 | 123, 212 | SSC | *ndhA(intron)* |
|  | T | 11 | 125, 627 | 125, 637 | SSC | *rps15* |
|  | T | 11 | 126, 427 | 126, 437 | SSC | *IGS(rps15,ycf1)* |
|  | A | 11 | 128, 244 | 128, 254 | SSC | *IGS(rps15,ycf1)* |
|  | T | 11 | 128, 828 | 128, 838 | SSC | *IGS(rps15,ycf1)* |
|  | A | 11 | 129, 467 | 129, 477 | SSC | *IGS(rps15,ycf1)* |
|  | A | 11 | 130, 121 | 130, 131 | SSC | *IGS(rps15,ycf1)* |
|  | T | 10 | 130, 300 | 130, 309 | IRA | *IGS(rps15,ycf1)* |
|  | T | 10 | 130, 419 | 130, 428 | IRA | *ycf1* |
|  | A | 10 | 130, 582 | 130, 591 | IRA | *ycf1* |
|  | T | 18 | 132, 907 | 132, 924 | IRA | *IGS(ycf1,trnN-GUU)* |
|  | G | 10 | 142, 976 | 142, 985 | IRA | *IGS(ycf15,rps7)* |
| **Dimer** | TA | 10 | 20, 506 | 20, 515 | LSC | *rpoC2* |
|  | AT | 10 | 29, 045 | 29, 054 | LSC | *IGS(trnC-GCA,petN)* |
|  | AT | 12 | 32, 243 | 32, 254 | LSC | *IGS(trnE-UUC,trnT-GGU)* |
|  | TA | 10 | 47, 798 | 47, 807 | LSC | *IGS(rps4,trnT-UGU)* |
|  | TA | 14 | 78, 842 | 78, 855 | LSC | *petD(intron)* |
|  | AT | 10 | 119, 162 | 119, 171 | SSC | *ndhD* |
| **Trimer** | AGA | 12 | 61, 331 | 61, 342 | LSC | *IGS(accD,psaI)* |
| **Tetramer** | AATT | 16 | 37, 534 | 37, 549 | LSC | *IGS(psbZ,trnG-UCC)* |
|  | TAGT | 12 | 60, 940 | 60, 951 | LSC | *IGS(accD,psaI)* |
|  | TCTA | 12 | 93, 953 | 93, 964 | IRB | *ycf2* |
|  | ATAG | 12 | 148, 538 | 148, 549 | IRA | *ycf2* |

Note: IGS, intergenic spacer.

**Table S5**. Details regarding the chloroplast genome sequences used for the phylogenetic analysis.

| No. | | Taxon | | Family | | Order | | GenBank Accession number | |
| --- | --- | --- | --- | --- | --- | --- | --- | --- | --- |
| 1 | | *Zanthoxylum piperitum* | | Rutaceae | | Sapindales | | KT153018 | |
| 2 | | *Zanthoxylum schinifolium* | | Rutaceae | | Sapindales | | NC_030702 | |
| 3 | | *Zanthoxylum simulans* | | Rutaceae | | Sapindales | | NC_037482 | |
| 4 | | *Citrus aurantiifolia* | | Rutaceae | | Sapindales | | NC_024929 | |
| 5 | | *Citrus platymamma* | | Rutaceae | | Sapindales | | NC_030194 | |
| 6 | | *Ailanthus altissima* | | Simaroubaceae | | Sapindales | | MG799542 | |
| 7 | | *Carapa guianensis* | | Meliaceae | | Sapindales | | NC_037442 | |
| 8 | | *Cedrela odorata* | | Meliaceae | | Sapindales | | NC_037251 | |
| 9 | | *Entandrophragma cylindricum* | | Meliaceae | | Sapindales | | NC_037250 | |
| 10 | | *Spondias bahiensis* | | Anacardiaceae | | Sapindales | | NC_030526 | |
| 11 | | *Spondias mombin* | | Anacardiaceae | | Sapindales | | NC_035973 | |
| 12 | | *Spondias tuberosa* | | Anacardiaceae | | Sapindales | | NC_030527 | |
| 13 | | *Boswellia sacra* | | Burseraceae | | Sapindales | | NC_029420 | |
| 14 | | *Dipteronia dyeriana* | | Sapindaceae | | Sapindales | | NC_031899 | |
| 15 | | *Dipteronia sinensis* | | Sapindaceae | | Sapindales | | NC_029338 | |
| 16 | | *Dimocarpus longan* | | Sapindaceae | | Sapindales | | NC_037447 | |
| 17 | | *Acer buergerianum* | | Acereae | | Sapindales | | NC_034744 | |
| 18 | | *Acer davidii* | | Acereae | | Sapindales | | NC_030331 | |
| 19 | | *Acer griseum* | | Acereae | | Sapindales | | NC_034346 | |
| 20 | | *Acer miaotaiense* | | Acereae | | Sapindales | | NC_030343 | |
| 21 | | *Acer morrisonense* | | Acereae | | Sapindales | | KT970611 | |
| 22 | | *Acer truncatum* | | Acereae | | Sapindales | | MH638284 | |
| *23* | | *Euonymus hamiltonianus* | | Celastraceae | | Celastrales | | NC_037518 | |

**Table S6.** The sequences variability of sequenced fragments.

|  | | Poly (T) repeat | | | | Delete | | | | | | | | | | Insert | |
| --- | --- | --- | --- | --- | --- | --- | --- | --- | --- | --- | --- | --- | --- | --- | --- | --- | --- |
| *A. tonkinense* | | (T)8 | | (T)5 | | T | | - | | CT | | T | | - | | - | |
| *A. ginnala* | | (T)9 | | (T)7 | | - | | AA | | CTT | | AATGGG | | - | | - | |
| 1. *negundo* | | (T)10 | | (T)5 | | TT | | - | | CT | | - | | TT | | - | |
| *A. henryi* | | (T)9 | | (T)4 | | T | | - | | - | | - | | T | | - | |
| *A. truncatum* | | (T)11 | | (T)3 | | - | | - | | CTT | | - | | - | | ATCAAAGG | |
| *A.buergerianum* | | (T)8 | | (T)4 | | - | | - | | C | | - | | - | | - | |
